# Non-redundant metagenome-assembled genomes of activated sludge reactors at different disturbances and scales

**DOI:** 10.1038/s41597-024-03601-9

**Published:** 2024-08-09

**Authors:** Soheil A. Neshat, Ezequiel Santillan, Hari Seshan, Stefan Wuertz

**Affiliations:** 1 Singapore Centre for Environmental Life Sciences Engineering, Nanyang Technological University Singapore, Singapore, 637551 Singapore; 2 Department of Civil and Environmental Engineering, University of California, Davis, California 95616 USA; 3 School of Civil and Environmental Engineering, Nanyang Technological University Singapore, Singapore, 639798 Singapore

**Keywords:** Metagenomics, Microbial ecology

## Abstract

Metagenome-assembled genomes (MAGs) are microbial genomes reconstructed from metagenomic data and can be assigned to known taxa or lead to uncovering novel ones. MAGs can provide insights into how microbes interact with the environment. Here, we performed genome-resolved metagenomics on sequencing data from four studies using sequencing batch reactors at microcosm (~25 mL) and mesocosm (~4 L) scales inoculated with sludge from full-scale wastewater treatment plants. These studies investigated how microbial communities in such plants respond to two environmental disturbances: the presence of toxic 3-chloroaniline and changes in organic loading rate. We report 839 non-redundant MAGs with at least 50% completeness and 10% contamination (MIMAG medium-quality criteria). From these, 399 are of putative high-quality, while sixty-seven meet the MIMAG high-quality criteria. MAGs in this catalogue represent the microbial communities in sixty-eight laboratory-scale reactors used for the disturbance experiments, and in the full-scale wastewater treatment plant which provided the source sludge. This dataset can aid meta-studies aimed at understanding the responses of microbial communities to disturbances, particularly as ecosystems confront rapid environmental changes.

## Background & Summary

Genome-resolved metagenomics and metagenome-assembled genomes (MAGs) have emerged as valuable tools in the study of microbial communities in a variety of systems and on different scales^1–3^. Activated sludge systems are widely employed in wastewater treatment, where a diverse array of microorganisms work synergistically to break down organic pollutants^1,4^. MAGs, generated from metagenomic data, can provide a comprehensive view of the genomic content of entire microbial communities within these systems^1,5,6^. They enable the identification and characterization of individual microbial species, shedding light on their potential metabolic capabilities, ecological roles, and interactions within the complex microbial network of activated sludge^7–9^.

Furthermore, the investigation of MAGs can play an important role when evaluating the responses of microbial communities to environmental fluctuations and disturbances at different scales^3^. Activated sludge systems often face variations in influent composition, temperature, and other operational parameters, which can impact microbial community dynamics and process performance^10–15^. By analysing MAG datasets, researchers can gain insights into the genomic adaptations that allow microbial communities to withstand such fluctuations. Meta-studies employing MAGs can contribute to our understanding of the resilience and functional redundancy within microbial communities^16,17^. The latter could be valuable not only for optimizing activated sludge systems to enhance wastewater treatment performance but also for broader applications in community ecology such as testing different theories and hypotheses^10,18^ and developing conceptual frameworks^19,20^. Furthermore, it can aid in understanding the responses of microbial communities to disturbances, particularly as global ecosystems face rapid environmental changes due to anthropogenic activity^21^.

**Fig. 1:**
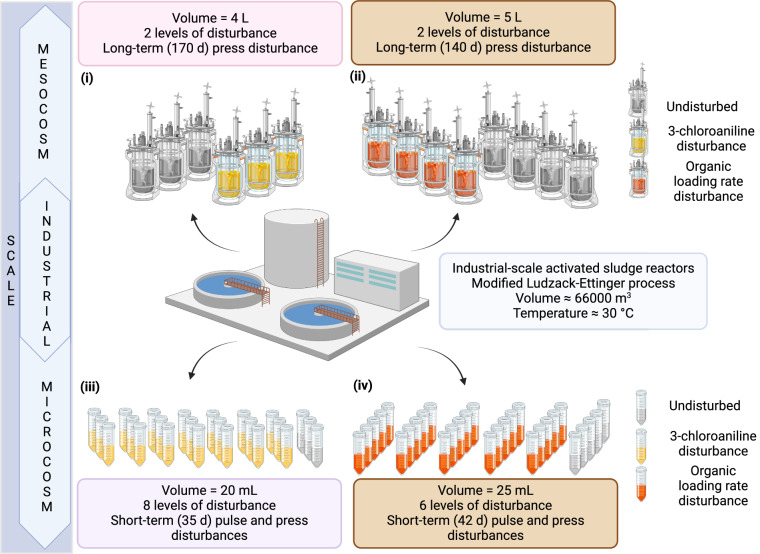
Disturbance types, scales, and experimental settings of the four disturbance experiments (numbered i - iv) conducted on activated sludge microbial ecosystems^10,11,16–18,20,22^. The total number of reactors and biological replicates for each experiment is presented, and reactors in each experiment are grouped based on the number of disturbance levels applied. A total of 114 samples were collected. The number of samples and their collection times for these experiments are as follows: (i) 6 from mesocosm-scale reactors (4 L) after 132 days under 2 levels of 3-CA disturbance; (ii) 44 from mesocosm-scale reactors (5 L) after 60 days under 2 levels of OLR disturbance, including 4 samples collected 1 day before any disturbance and 8 samples collected 14 days post-disturbance; (iii) 24 from microcosm-scale reactors (20 mL) after 35 days under 8 levels of 3-CA disturbance; (iv) 30 from microcosm-scale reactors (25 mL) after 42 days under 6 levels of OLR disturbance; and 10 from full-scale activated sludge reactors constituting three different sets of sludge inocula. Created with BioRender.com.

In this study, we present a collection of 839 MAGs derived from sequencing datasets that encompass 114 metagenomic samples collected from activated sludge reactors across four comprehensive disturbance experiments^10,11,16–18,20,22^. Utilizing sequencing batch reactors at microcosm (20 and 25 mL)^10,18,20^ and mesocosm (4 and 5 L)^11,16,17,22^ scales, and starting from full-scale sludge inocula, these experiments aimed to elucidate the responses of activated sludge wastewater treatment communities to environmental disturbances (Fig. 1). Wastewater treatment plants operate in real-world conditions and are expected to function under various circumstances, such as the accidental introduction of toxic chemicals into the waste streams, as well as fluctuations in inflow rates and concentrations due to heavy rain or incidental discharges. Hence, the disturbances in focus included the presence of the toxic compound 3-chloroaniline (3-CA)^10,16,18,22^ and variations in organic loading rate (OLR)^11,17,20^. Such disturbances are known to inhibit important functions in activated sludge performance and alter microbial community structure and assembly^10,11,16–18,20,22^. Beyond wastewater treatment, this dataset enables researchers to study the effects of various types of disturbances, their frequencies of exposure, and their impacts on different scales within complex microbial systems.

**Fig. 2:**
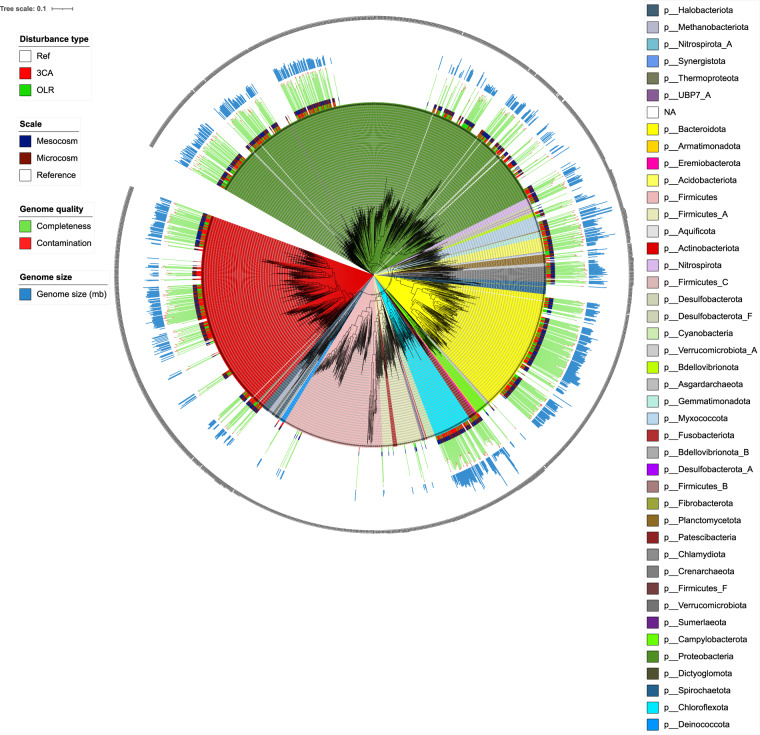
The phylogenetic tree of the constructed MAGs inserted into the tree of life using the PhyloPhlan database with the Phylophlan 3 software package. The interactive figure is available through the iTOL website. The tree information including branch lengths and bootstrap values can be extracted from the tree file. The scale of the study (first coloured strips), type of disturbance (second coloured strip), quality scores (completeness and contamination), and genome sizes of the MAGs are shown around the tree. No values are included for reference genomes or low-quality (completeness < 50% and contamination >10%) MAGs.

Bioinformatics analyses on these datasets yielded 1466 MAGs of which 839 (57%) met the medium-quality criteria of >50% completeness and <10% contamination defined by Bowers *et al*.^23^. Further quality assessment revealed 399 MAGs of putatively high-quality with a completeness and contamination scores of more than 90% and less than 5%, respectively. All these MAGs harboured more than 18 tRNA genes, but only 67 carried a full complement of 5S, 16S and 23S rRNA genes. The MAGs of at least medium quality were taxonomically classified as bacteria spanning across 23 phyla (Fig. 2). They were assigned to 43 classes, 86 orders, 135 families, 177 genera and 40 known species. Dereplication showed that there are 297 non-redundant MAGs at genus level in this collection. The most diverse phyla were *Proteobacteria*, *Actinobacteriota*, *Bacteroidota*, *Patescibacteria*, and *Chloroflexota* with 250, 174, 163, 70, and 57 assigned MAGs, respectively. Summary statistics of the MAGs in this catalogue including taxonomy classification and quality measures are presented in Supplementary Table 1.

The catalogue of MAGs enables a better understanding of microbial community dynamics and genomic adaptations under varied disturbance conditions and scales, thereby enhancing our broader knowledge of activated sludge reactor performance. These insights not only aid in the optimization of wastewater treatment processes but also contribute to broader ecological studies, offering a glimpse into the adaptability of microbial communities to environmental and anthropogenic disturbances.

## Methods

### Experimental design and sampling

#### Sludge inoculum collection

Sludge inoculum was collected on four different occasions at the beginning of each of the four experiments^10,11,16–18,20,22^, from one of the activated sludge tanks of a water reclamation plant in Singapore with a Modified Ludzack-Ettinger (MLE) process configuration. Operational parameters were: Flow rate ≈ 200,000 m^3^/d, temperature ≈ 30 °C, pH ≈ 6.7, total suspended solids (TSS) ≈ 1,500 mg/L, hydraulic retention time (HRT) = 8 h, and solids retention time (SRT) = 5 – 6 d. Typical influent concentrations were: total Kjeldahl nitrogen (TKN) ≈ 49 mg/L and total chemical oxygen demand (COD) ≈ 320 mg/L. The plant receives a mix of mostly residential, commercial and industrial wastewater as its influent, operating continuously at C:N ≈ 6.5 mg COD/mg TKN and food-to-biomass ratio (F:M) ≈ 0.21 mg COD/mg TSS/d. Activated sludge was collected in 20-L containers and immediately transported to the lab. The suspension was manually mixed by shaking the closed container thoroughly before transferring it to the reactors. Subsequently, aliquots of 2 mL were collected for DNA extraction to assess the initial (d1) microbial community from each study via metagenomics sequencing (except for the 3-CA mesocosm study which only had metabarcoding done on d1 samples). A total of ten samples of full-scale activated sludge tanks were retrieved, corresponding to sludge inocula from three different studies^10,11,17,18,20^.

#### 3-CA disturbance microcosm experiment

As described in Santillan *et al*.^10,18^, twenty-four sequencing batch reactors (SBRs) of 20-mL working volume were inoculated with activated sludge from the above-described full-scale plant and operated for 35 d. The complex synthetic feed included 3-CA at varying frequencies. Eight levels of disturbance were set in triplicate independent reactors (n = 3), which received 3-CA every day (press-disturbed), every two, three, four, five, six, or seven days (intermediately-disturbed), or never (undisturbed). Level numbers were assigned from 0 to 7 (0 for no disturbance, 1 to 7 for low to high disturbance frequency). All reactors were handled ensuring sterile conditions, capped, and operated in a shaking incubator at 30 °C. After each cycle (24 h) all the tubes were removed from the incubator and allowed to settle for 30 min, after which 10 mL of effluent supernatant liquid was removed and replaced aseptically with 10 mL of fresh synthetic medium, resulting in a 48-h HRT. Concentrations in the mixed liquor after feeding (*i.e*., beginning of a new cycle) were of 590 (± 15.4) mg COD/L and 92 (± 2.5) mg N/L (in the form of ammonium compounds). The medium for disturbed levels also included 140 mg/L of 3-CA that resulted in 70 mg/L in the mixed liquor. Phosphates were used to buffer the medium and maintain a pH of around 7.5 to facilitate the nitrification process. On the final day of the experiment, all the remaining sludge in the reactors was employed for gravimetric biomass measurements. The sludge collection scheme resulted in an SRT of 70 d. Sludge samples were collected on d1 (2 samples, 2 mL each, taken at random from the inoculum mix) and d35 (24 samples, 1 mL from each reactor), and stored at −80 °C for DNA extraction and metagenomics sequencing.

#### 3-CA disturbance mesocosm experiment

Six SBRs of 4-L working volume were inoculated with activated sludge from the above-described full-scale wastewater treatment plant as described in Seshan *et al*.^16^ and Santillan *et al*.^22^. The reactors were run in parallel and fed on synthetic wastewater for a 58-d acclimation period. The stages of each 12-h cycle were as follows: 20 min feeding, 190 min anoxic mixing, 440 min aeration (dissolved oxygen, DO, maintained at 1-2 mg/L using a feedback loop with aeration beginning at 1 L air/min when probes measured DO < 1 mg/L, and stopping when DO > 2 mg/L), 50 min settling and 30 min effluent (supernatant) discharge. Two liters of effluent were discharged at the end of each cycle and replaced with 2 L of synthetic wastewater at the beginning of the next 12 h cycle, resulting in a 24 h HRT. The mixed liquor temperature was maintained at 30 °C using water jackets around the reactors and a re-circulating water heater. Solids were removed regularly from the mixed liquor to maintain an SRT of about 30 d. Concentrations in the mixed liquor after feeding (*i.e*., beginning of a new cycle) were about 500 mg COD/L and 70 mg N/L (ammonium-based compounds). The medium for disturbed levels included 140 mg/L of 3-CA that resulted in 70 mg/L in the mixed liquor. In the treatment reactors, six macro-components in the synthetic wastewater recipe were scaled down by 20% to reduce their contribution to COD to 400 mg/L. The remaining COD was fed in the form of 3-CA, to a total of 100 mg/L as COD (~65 mg/L as 3-CA). Phosphates were used to buffer the medium and maintain a pH of around 7.5 to facilitate nitrification. The 3-CA press disturbance experiment started on d59 and continued for 132 d, for total study period of 190 d. At the beginning of this stage, three reactors were randomly assigned to the treatment group and the other three were assigned to the control group. Cycle conditions and other parameters were identical to those of the acclimation phase. The three control reactors continued to receive the same 3-CA-free medium used during acclimation. On d99, a pulse 3-CA load was inadvertently added to one of the control reactors labelled as C3 at the same concentration as the treatment reactors. Sludge samples were collected from all reactors on d176 (6 samples, 2 mL each) and stored at −80 °C for DNA extraction and metagenomics sequencing.

#### OLR disturbance microcosm experiment

Thirty SBRs of 25-mL working volume were inoculated with activated sludge from the above-described full-scale wastewater treatment plant and operated for 42 d as described in Santillan *et al*.^20^. The daily complex synthetic feeding regime included double organic loading at varying disturbance frequencies. Six levels of disturbance were set in quintuplicate independent reactors (n = 5), which received double organic loading either never (undisturbed), every eight, six, four, or two days (intermediately disturbed), or every day (press disturbed). Level numbers were assigned from 0 to 5 (0 for no disturbance, 1 to 5 for low to high disturbance frequency). All reactors were handled ensuring sterile conditions, capped and operated in a shaking incubator at 30 °C. After each cycle (24 h) all the tubes were removed from the incubator and allowed to settle for 30 min, after which 12.5 mL of effluent supernatant liquid was removed and replaced aseptically with 12.5 mL of fresh synthetic medium, resulting in a 48-h HRT. Concentrations in the mixed liquor of the bioreactors after feeding (*i.e*., beginning of a new cycle) were either 305.8 (± 7.4) mg COD/L and 45.6 (± 0.8) mg TKN/L, or 594.7 (± 18.6) mg COD/L and 46.1 (± 0.2) mg TKN/L when double organic loading occurred. Phosphate addition targeted a concentration in the mixed liquor of 7.45 (± 0.8) mg P/L to obtain an N:P of around 6. To control the F:M, biomass was measured weekly as TSS, after which sludge was wasted to target a TSS of 1,500 mg/L. The latter resulted in average SRT values of 30, 26, 23, 22, 19 and 15 d, for disturbance levels from 0 to 5, respectively. Sludge samples of 2 mL were collected on d1 (4 samples, taken at random from the inoculum mix) and d42 (30 samples, one per reactor), and stored at −80 °C for DNA extraction and metagenomics sequencing.

#### OLR disturbance mesocosm experiment

As described in Santillan *et al*.^11,17^, four SBRs of 5-L working volume were inoculated with activated sludge from the full-scale wastewater treatment plant. Reactors were first acclimated to lab conditions and fed with complex synthetic wastewater for 53 d. At the start of the disturbance experiment (d54), the sludge of the acclimation reactors was thoroughly mixed and redistributed across eight SBRs. From these, four were randomly selected and designated as high OLR reactors, receiving double the amount of organic carbon in terms of COD in their feed as a press disturbance for 60 d. The remaining four reactors were operated as before at low OLR. During the last two weeks of the study (d114-d127), the feed for the high OLR reactors was adjusted to equal that of the low-OLR reactors. As one of the high-OLR reactors experienced an operational issue (air diffuser blockage on d64), data from this reactor were not included in Santillan *et al*.^11^, reducing the total number of replicates for the high OLR group to three in those studies. However, the performance and community dynamics of this reactor were detailed in Santillan^24^, and its metagenomics data were also included for the analysis in the present work. Concentrations in the mixed liquor of the bioreactors after feeding (*i.e*., beginning of a new cycle) were either 323 (± 24) mg COD/L and 92 (± 3.6) mg TKN/L, or 629 (± 67) mg COD/L and 100 (± 19) mg TKN/L when double organic loading occurred. The water temperature in the reactor was maintained at 30 °C, and sludge was continuously mixed with a magnetic stirrer. Each SBR cycle consisted of 5 min feeding, 200 min anoxic/anaerobic reaction, 445 min aerobic reaction, 50 min sludge settling, and 20 min supernatant draining. The DO concentration was maintained at 2–6 mg/L during the aerobic phase. The pH ranged from 6 to 9, owing to alkalinity provided in the feed. After sludge settling, 2.5 L of the supernatant effluent was discharged, followed by the replacement of the same volume with synthetic wastewater during the feeding phase at the beginning of the next 12-h cycle. Two cycles per day corresponded to an HRT of 24 h. To control the F:M, sludge biomass was measured as TSS twice a week, after which enough sludge was wasted to maintain a TSS of 1,500 mg/L. This resulted in average SRT values of 7.9 and 5.1 d for low and high OLR reactors, respectively. Sludge aliquots of 2 mL were collected from all four reactors on d1 (inoculum) and d53 of the acclimation phase (8 samples), and from all eight reactors during the press disturbance phase on days 56, 75, 96, 110 and 124 (40 samples). These aliquots were stored at −80 °C for DNA extraction and metagenomics sequencing.

#### Nucleic acid extraction and whole-genome sequencing

DNA extraction was performed using the MP Biomedicals FastDNA® spin kit for soil (MP Biomedicals, Irvine, CA, USA) following the manufacturer’s instruction. The extracted DNA was further purified and concentrated using ZymoBIOMICS DNA Clean & Concentrator (Zymo Research, Irvine, CA, USA) prior to library preparation. The quality, quantity and integrity of the extracted DNA was measured using a Nanodrop spectrophotometer (Thermo Fisher Scientific, Waltham, MA, USA), Qubit2 Fluorometer (Thermo Fisher Scientific, Waltham, MA, USA) and Agilent Tapestation (Agilent technologies, Santa Clara, CA, USA), respectively. Sequencing libraries were prepared employing the Illumina TruSeq Nano DNA (Illumina, San Diego, CA, USA) sample preparation protocol. Sequencing was performed on an Illumina HiSeq. 2500 sequencing machine (Illumina, San Diego, CA, USA) with a read length and sequencing mode of 250 bp and paired-end mode, respectively. The raw sequences are available through the NCBI sequence read archive with the BioProjects accession codes 389377^25^, 559245^26^, 720805^27^ and 723443^28^. The raw sequencing files were generated within the context of previously published studies by Santillan *et al*.^10,11,16–18,20,22^.

### Bioinformatics pipeline

#### Sequencing reads quality assessment and trimming

The generated sequences were trimmed to remove sequencing adapters and low-quality reads using Trimmomatic v0.36^29^ (non-default parameters: ILLUMINACLIP: adapters.fa:2:30:10 LEADING:3 TRAILING:3 SLIDINGWINDOW:4:14 MINLEN:36). This step ensured that only reads meeting a quality score of at least Q30 were retained for assembly. Fastqc^30^ was used to check the quality of the reads before and after trimming.

#### Read assembly

Co-assemblies were performed on replicate/time series samples taken from reactors in the same experiments (including control reactors) using SPAdes^31^ v3.13.0 using kmer sizes of 21, 33, 55, 77, 99, and 121 in metagenomics mode (parameters:–meta -t 72 -m 1500 -k 21,33,55,77,99,121).

#### Scaffolds binning and genome bin dereplication

The raw reads were mapped into the assembled contigs using BBMAP^32^ software package to generate the coverage profiles. Binning was performed using the Metabat2^33^ v2.12.1 software package with default parameters. The recovered genome-bins were then pooled and dereplicated using the dRep pipeline^34^ with default parameters except for -p 64 -comp 50 -con 10 -nc 0.5 -pa 0.9 -sa 0.95 to select species level non-redundant MAGs with at least medium quality according to MIMAG criteria^23^. The dRep pipeline uses CheckM^35^ v1.0.13 for quality assessment, mesh v2.2 for distance estimation, and Prodigal^36^ v2.6.3 for open reading frame calling.

#### Taxonomy classification

Taxonomy classification was carried out by GTDB-Tk v1.7.0^37,38^ software package with database release 214 using the classify workflow (classify_wf) with default parameters.

#### Genome bin quality assessment

Quality of the genome bins were assessed using CheckM v1.0.13 lineage workflow (lineage_wf) with default parameters. The extended report was generated using the CheckM qa pipeline with the flag -o 2. The rRNA and tRNA annotations were performed using RNA check^39^ utilising barrnap^40^ v0.9 and tRNAscan-SE^41^ v2.0.12.

#### Open reading frame (ORF) annotation and functional analysis

The ORF calling was performed using Prokka^42^ v1.13. Comprehensive functional analysis of the recovered MAGs was performed using EggNOG mapper^43^ v2.1.2.

#### Relative abundance calculations

The relative abundance of MAGs in samples was calculated using CoverM^44^ v0.6.1. For this analysis, a genome workflow was utilised with the following parameters,–min-covered-fraction 5, and–genome-fasta-extension fa. In addition, the trimmed reads and the pool of MAGs were used as the input for CoverM.

## Data Records

A set of dereplicated MAGs at genus level is available through the NCBI GenBank with the BioProject accession number of 1089772^45^. The recovered MAGs dereplicated at strain level in fasta format (AS_bins.tar.gz), functionally annotated genomes (AS_MQ_functional_annotation.tar.gz), relative abundance the MAGs across samples (AS_MQ_bins_relative_abundance.tar.gz), and the phylogenetic tree files (Phylogenetic_tree.tre and AS_D_MAGs.svg) are available through Zenodo^46^ with digital object identifier of 10.5281/zenodo.8405311. The summary statistics of the MAGs along with taxonomy classification and CheckM extended quality measures are available in Supplementary Table 1.

## Technical Validation

The draft genomes included in this manuscript meet the medium quality requirements based on the MIMAG criteria^23^.

## Supplementary information

TABLE 1

## Data Availability

No custom codes were used for this analysis.
