# Author Correction: Non-redundant metagenome-assembled genomes of activated sludge reactors at different disturbances and scales

**DOI:** 10.1038/s41597-024-03957-y

**Published:** 2024-10-11

**Authors:** Soheil A. Neshat, Ezequiel Santillan, Hari Seshan, Stefan Wuertz

**Affiliations:** 1 Singapore Centre for Environmental Life Sciences Engineering, Nanyang Technological University Singapore, Singapore, 637551 Singapore; 2 Department of Civil and Environmental Engineering, University of California, Davis, California 95616 USA; 3 School of Civil and Environmental Engineering, Nanyang Technological University Singapore, Singapore, 639798 Singapore

**Keywords:** Metagenomics, Microbial ecology, Metagenomics, Microbial ecology

Correction to: *Scientific Data* 10.1038/s41597-024-03601-9, published online 9 August 2024

In the version of the article initially published, “Created with BioRender.com” was missing from the caption to Fig. 1 and ref. 46 included the wrong DOI so has now been amended to “Neshat, S., Santillan, E., Seshan, H. & Wuertz, S. Non-redundant metagenome-assembled genomes of activated sludge reactors at different disturbances and scales, *Zenodo*, 10.5281/zenodo.8405311 (2023)”. These corrections have been made to the HTML and PDF versions of the article.

